# Monitoring serum potassium concentration in patients with severe hyperkalemia: the role of bloodless artificial intelligence-enabled electrocardiography

**DOI:** 10.1093/ckj/sfaf092

**Published:** 2025-04-08

**Authors:** Chien-Chou Chen, Chin Lin, Ding-Jie Lee, Chin-Sheng Lin, Sy-Jou Chen, Chih-Chien Sung, Yu-Juei Hsu, Shih-Hua Lin

**Affiliations:** Division of Nephrology, Department of Medicine, Tri-Service General Hospital, National Defense Medical Center, Taipei, Taiwan, R.O.C; Division of Nephrology, Department of Medicine, Tri-Service General Hospital Songshan branch, National Defense Medical Center, Taipei, Taiwan, R.O.C; Graduate Institute of Life Sciences, National Defense Medical Center, Taipei, Taiwan, R.O.C; School of Medicine, National Defense Medical Center, Taipei, Taiwan, R.O.C; Division of Nephrology, Department of Medicine, Tri-Service General Hospital, National Defense Medical Center, Taipei, Taiwan, R.O.C; Division of Cardiology, Department of Internal Medicine, Tri-Service General Hospital, National Defense Medical Center, Taipei, Taiwan, R.O.C; Department of Emergency Medicine, Tri-Service General Hospital, National Defense Medical Center, Taipei, Taiwan, R.O.C; Division of Nephrology, Department of Medicine, Tri-Service General Hospital, National Defense Medical Center, Taipei, Taiwan, R.O.C; Division of Nephrology, Department of Medicine, Tri-Service General Hospital, National Defense Medical Center, Taipei, Taiwan, R.O.C; Division of Nephrology, Department of Medicine, Tri-Service General Hospital, National Defense Medical Center, Taipei, Taiwan, R.O.C

**Keywords:** artificial intelligence, deep learning, electrocardiography, hyperkalemia, monitor

## Abstract

**Background:**

Severe hyperkalemia is a life-threatening emergency requiring prompt management and close surveillance. Although artificial intelligence-enabled electrocardiography (AI-ECG) has been developed to rapidly detect hyperkalemia, its application to monitor potassium (K^+^) levels remains unassessed. This study aimed to evaluate the effectiveness of AI-ECG for monitoring K^+^ levels in patients with severe hyperkalemia.

**Methods:**

This retrospective study was performed at an emergency department of a single medical center over 2.5 years. Patients with severe hyperkalemia defined as Lab-K^+^ ≥6.5 mmol/l with matched ECG-K^+^ ≥5.5 mmol/l were included. ECG-K^+^ was quantified by ECG12Net analysis of the AI-ECG system. The following paired ECG-K^+^ and Lab-K^+^ were measured at least twice, almost simultaneously, during and after K^+^-lowering therapy in 1 day. Clinical characteristics, pertinent intervention, and laboratory data were analyzed.

**Results:**

Seventy-six patients fulfilling the inclusion criteria exhibited initial Lab-K^+^ 7.4 ± 0.7 and ECG-K^+^ 6.8 ± 0.5 mmol/l. Most of them had chronic kidney disease (CKD) or were on chronic hemodialysis (HD). The followed Lab-K^+^ and ECG-K^+^ measured with a mean time difference of 11.4 ± 5.6 minutes significantly declined in parallel both in patients treated medically (*n* = 39) and with HD (*n* = 37). However, there was greater decrement in Lab-K⁺ (mean 7.3 to 4.1) than ECG-K⁺ (mean 6.6 to 5.0) shortly after HD. Three patients with persistent ECG-K^+^ hyperkalemia despite normalized Lab-K^+^ exhibited concomitant acute cardiovascular comorbidities.

**Conclusions:**

AI-ECG for K^+^ prediction may help monitor K^+^ level for severe hyperkalemia and reveal more severe cardiac disorders in the patients with persistent AI-ECG hyperkalemia.

## Graphical Abstract

**Graphical Abstract:**
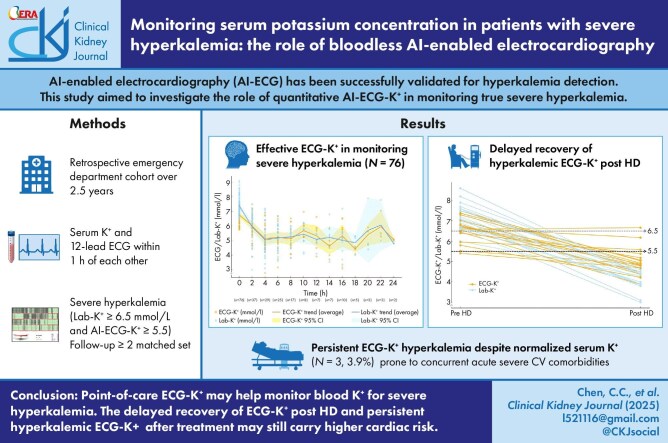

KEY LEARNING POINTS **What was known**:

- AI-enabled echocardiography (AI-ECG) has been successfully validated for hyperkalemia detection.
- Although AI-ECG has been used to rule out hyperkalemia, its application in monitoring true severe hyperkalemia during hyperkalemia treatment remains unexplored.

**This study adds**:

- The reduction in quantitative AI-ECG-K^+^ (ECG-K^+^) during treatment of severe hyperkalemia was significantly parallel to Lab-K^+^.
- The delayed recovery of hyperkalemic ECG-K^+^ post-HD, and persistent ECG-K^+^ hyperkalemia despite normalized Lab-K⁺ in patients experiencing acute cardiovascular events suggest that hyperkalemic ECG-K^+^ may still carry a higher cardiac risk.

**Potential impact**:

- In addition to early detection of severe hyperkalemia, AI-ECG may be alternatively used to monitor serum K^+^ changes during hyperkalemia treatment. Given its potential capability, AI-ECG can facilitate the development of personalized prevention and treatment strategies for severe hyperkalemia and its associated cardiovascular complications.

## INTRODUCTION

Hyperkalemia, which elevates the risk of cardiovascular morbidity and mortality, occurs in ∼3% of emergency department (ED) admissions. This condition is even more prevalent among patients with advanced chronic kidney disease (CKD) or those experiencing acute kidney failure and oliguria [1, 2]. Severe hyperkalemia (usually defined as serum potassium (K^+^) level ≥6.5 mmol/l) is particularly life-threatening with potentially fatal arrhythmias and cardiac death, requiring prompt recognition with a swift therapy and surveillance [3, 4]. As K^+^ is essential for cardiac tissue functionality [5, 6], alterations in the heart's electrical activity influenced by varying degrees of hyperkalemia, may be discerned through electrocardiogram (ECG) analysis [7, 8]. To detect severe hyperkalemia and monitor the effect of hyperkalemia treatment, rapid measurement of blood K^+^ level and ECG changes with frequent monitoring are absolutely warranted to avoid cardiac arrhythmia [9–11]. Nevertheless, the ECG changes associated with severe hyperkalemia cannot be easily identified even with experienced clinicians [12–14].

With the advent of deep learning models (DLM), artificial intelligence (AI)-enabled ECG systems (AI-ECG) have been successfully developed and validated [15–21]. For severe hyperkalemia detection with serum K^+^ level ≥6.5 mmol/l, our AI-ECG system using 12-lead ECG analysis achieved higher areas under the curve of 0.966 with a sensitivity of 93.8% [16, 21]. Most importantly, the AI-ECG-K⁺ system was meticulously designed to function on a continuous predictive scale rather than adopting a binary classification model, thereby facilitating individualized quantification to the severity of hyperkalemic ECG changes [16, 21].

During the management of severe hyperkalemia, it is imperative to evaluate treatment effectiveness through quantitative methodologies and adjust strategies in case of inadequate response. Although repeated serum K^+^ measurements with varying turnaround times have traditionally been required to evaluate treatment response, the quantified AI-ECG-K^+^ provides real-time predictions of serum K^+^ levels and may offer potential benefits. Nevertheless, the application of AI-ECG-K^+^ to monitor K^+^ levels during the management of true severe hyperkalemia has not been evaluated.

In this 2.5-year retrospective cohort study, we assessed the utility of AI-ECG-derived K^+^ levels (ECG-K^+^) in the patients with severe hyperkalemia receiving at least two concurrent recordings of ECG-K^+^ and laboratory-measured levels (Lab-K^+^) during and after hyperkalemia treatment.

## MATERIALS AND METHODS

This study spanning from the ED to inpatient care at a single medical center (Tri-Service General Hospital in Taipei, Taiwan) was undertaken with ethical approval from the institutional review board (IRB NO. B202205054). We waived the patients’ consent owing to retrospectively collecting data in encrypted files from the hospital data controller.

### Patient selection

Adult patients aged 18 and older who were assessed with both Lab-K^+^ and ECG-K^+^ at the ED between 1 February 2019 and 31 August 2021, were initially enrolled. We first identified the patients with severe hyperkalemia defined by a Lab-K^+^ ≥6.5 mmol/l and a concurrent AI-ECG-K^+^ test within 1 hour. As normal 12-lead ECGs, which have been frequently reported in patients with severe hyperkalemia, could not be used for monitoring hyperkalemia [13, 22], we excluded the patients with severe hyperkalemia who had an initially matched ECG-K^+^ <5.5 mmol/l. To evaluate the effect of ECG-K^+^ more precisely, only patients who had at least two nearly simultaneous matches between ECG-K^+^ and Lab-K^+^ during and after hyperkalemia treatment within 1 day were ultimately included. All clinical characteristics, including kidney-specific comorbidities such as advanced CKD (aCKD), end-stage kidney disease on dialysis (ESKDd), acute kidney injury (AKI), and acute-on-CKD (AoCKD) as defined by the Kidney Disease Improving Global Outcomes (KDIGO) criteria [23], were retrieved from electronic medical records. In addition, information on hyperkalemia-associated medications, treatment modalities, laboratory data, and echocardiographic findings were also collected and analyzed.

### Measurement of Lab-K^+^ and ECG-K^+^

Lab-K^+^ was serum K^+^ concentration determined by International Organization for Standardization (ISO) certified central laboratory, with the exclusion of pseudohyperkalemia confirmed by both technicians and clinical physicians. The time nearest relevant laboratory data were assigned to each Lab-K^+^ record. ECGs were captured using a Philips 12-lead ECG machine (Model PH080A), generating a 5000-point digital sequence with a sampling rate of 500 Hz over a duration of 10 s per lead. ECG-K^+^ levels were computed by ECG12Net, an established DLM with 82 convolutional layers, which analyzed morphological variations instead of relying on traditional ECG morphology parameters such as heart rate, PR interval, QRS duration, QT interval, corrected QT (QTc), P wave axis, RS wave axis, or T wave axis, achieving a high sensitivity of 93.8% and specificity of 91.8% for serum K^+^ levels ≥6.5 mmol/l on hyperkalemia detection [21]. This DLM was trained on >40 000 samples and validated on an independent test set comprising >10 000 samples for over 5 years from 2011 to 2016. These patients were stratified into training, validation, and test sets, constituting ∼70%, 10%, and 20% of the cohort, respectively, based on their presentation dates throughout the study period [16].

As shown in Supplementary Fig. S1, the AI-ECG based analysis visualized hyperkalemia in a 12-lead ECG format, with each lead providing a weighted prediction of the hyperkalemia value. Based on the established training set, the reported K^+^ levels of AI-ECG were confined to a range of 1.5 to 7.5 mmol/l. AI-ECG-K^+^ ≥5.5 mmol/l was defined as ECG-K^+^ hyperkalemia as previously reported [16, 21]. We also defined the degree of laboratory hyperkalemia as mild hyperkalemia (Lab-K^+^ 5.5–5.9 mmol/l), moderate hyperkalemia (Lab-K^+^ 6.0–6.4 mmol/l), and severe hyperkalemia (Lab-K^+^ ≥6.5 mmol/l).

### Lab-K^+^ and ECG-K^+^ during and after hyperkalemia treatment

The hyperkalemia treatment included intravenous calcium gluconate or chloride (CaCl_2_) for myocardial protection, insulin, and/or inhaled β_2_-agonists to translocate serum K^+^ into cells, loop diuretics to enhance renal K^+^ excretion and even oral K^+^-chelating agents (calcium polystyrene sulfonate). The dialysate K^+^ concentration 1.0 mmol/l was used for the treatment of severe hyperkalemia. Owing to the variability in the timing of paired Lab-K⁺ and ECG-K⁺ measurements, the length of detailed time information during hyperkalemia treatment within 1 day was illustrated. We also used relative timepoints for these pairings, with time 1 designated for pre-treatment K^+^ levels and time 2–4 for post-treatment K^+^ levels collectively within a 24-hour period.

### Statistical analysis

The patients’ characteristics were presented as means and standard deviations or percentages where appropriate. The ECG-K^+^ and Lab-K^+^ before, during, and after hyperkalemia treatment were shown as mean values and 95% confidence interval (CI). The Bland and Altman plots were also constructed to show the mean differences and 95% CI between ECG-K^+^ and Lab-K^+^. The paired Student's *t*-test, chi-square test, and repeated measures analysis of variance test were used to assess the difference. The statistical analysis was conducted using the software environment R v.3.4.4. *P* < .05 was considered as significant.

## RESULTS

### Patients’ characteristics

**Figure 1:**
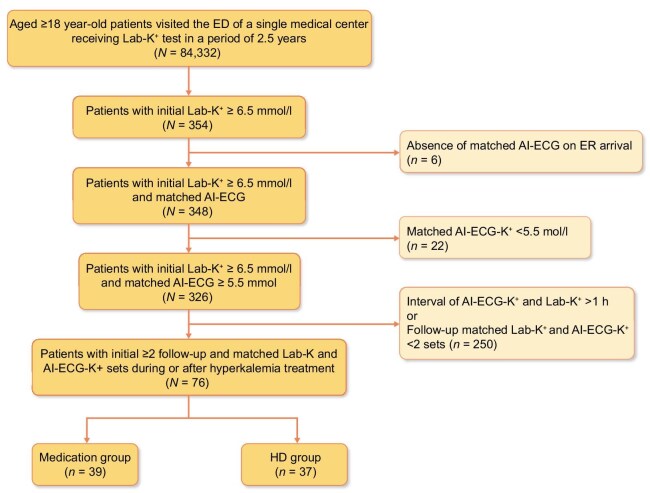
Flowchart of the study. Algorithm for the included patients with severe hyperkalemia.

As shown in Fig. 1, 354 out of 84 332 (0.4%) patients visiting the ED had severe hyperkalemia (Lab-K^+^ ≥6.5 mmol/l), and 326 of them had concurrent ECG-K^+^ ≥ 5.5 mmol/l. With the exclusion of unmatched AI-ECG-K^+^ and Lab-K^+^ measurement and the matched ECG-K^+^ and Lab-K^+^ fewer than two sets, only 76 patients fulfilled the inclusion criteria eventually. Their initial mean Lab-K^+^ and ECG-K^+^ were 7.4 (95% CI 7.3–7.6) and 6.8 (95% CI 6.6–6.9) mmol/l, respectively, with a mean time difference of 10.6 ± 5.1 min. These patients received either medical treatment only (medication group, *n* = 39) or HD therapy with initial medical treatment (HD group, *n* = 37). All 76 patients had received intravenous calcium salt and insulin as initial hyperkalemia treatment.

Their kidney-specific disorders included advanced CKD (aCKD) (*n* = 23), end-stage kidney disease under dialysis (ESKDd) (*n* = 28), AKI (*n* = 3), and AoCKD (*n* = 22). They also featured advanced age, higher portion of hypertension (*n* = 64), diabetes mellitus (DM) (*n* = 41), coronary artery disease (*n* = 37), hyperlipidemia (*n* = 23), and chronic heart failure (*n* = 21). Approximately half of them received chronic drug use such as angiotensin-converting enzyme inhibitors, angiotensin receptor blockers, or β-blockers, and diuretics including K^+^-sparing agents. Their laboratory findings were notable for anemia, metabolic acidosis, hypoalbuminemia, elevated serum pro-B type natriuretic peptide (proBNP), cardiac enzymes, C-reactive protein (CRP), and procalcitonin level. Moreover, the patients receiving HD had significantly higher percentage of aCKD and ESKDd, higher serum proBNP, CRP, and lower albumin level, as compared to those treated with medication only (Table 1).

**Table 1:** Clinical characteristics of patients with paired AI-ECG and laboratory hyperkalemia.

	Medication group (*n* = 39)	HD group (*n* = 37)	*P* value
AI-ECG-K^+^ (ref range 3.6–5.4, mmol/l)	6.7 ± 0.5	6.8 ± 0.5	.558
Lab-K^+^ (ref. range 3.6–5.4, mmol/l)	7.4 ± 0.6	7.4 ± 0.7	.879
Demography			
Age (years)	78.1 ± 13.8	73.0 ± 14.7	.126
Female	20 (51.3%)	13 (35.1%)	.160
BMI	23.3 ± 5.2	23.4 ± 3.6	.550
SBP (mmHg)	127.0 ± 25.4	126.9 ± 29.9	.986
General comorbidities			
DM	16 (41.0%)	25 (67.6%)	.020
HTN	32 (82.1%)	32 (86.5%)	.602
HLP	10 (25.6%)	13 (35.1%)	.375
CAD	16 (41.0%)	21 (56.8%)	.175
Stroke	6 (15.4%)	6 (16.2%)	.922
CHF	8 (20.5%)	13 (35.1%)	.158
COPD	1 (2.6%)	2 (5.4%)	.531
Kidney-specific comorbidities			
aCKD	19 (48.7%)	4 (10.8%)	<.001
ESKDd	7 (17.9%)	21 (56.8%)	<.001
AKI	3 (7.7%)	0 (0%)	.160
AoCKD	10 (25.6%)	12 (32.4%)	.513
Chronic drug use			
ACEI/ARB	17 (43.6%)	22 (59.4%)	.167
MRA	10 (25.6%)	9 (24.3%)	.896
ß-blockers	19 (48.7%)	23 (62.2%)	.166
Laboratory data (ref range)			
WBC (4.5–11.0) (10^3^/µl)	13.4 ± 8.9	10.1 ± 3.9	.046
Hb (M: ∼13.5–18.0, F: ∼12.0–16.0) (gm/dl)	9.9 ± 3.0	9.1 ± 2.0	.176
PLT (150–400) (10^3^/µl)	223.8 ± 97.7	196.5 ± 101.1	.239
pH (7.35–7.45)	7.2 ± 0.2	7.212 ± 0.162	.879
HCO_3_^−^ (23–27) (mmol/l)	16.9 ± 6.3	14.8 ± 6.8	.349
Na^+^ (135–145) (mmol/l)	133.3 ± 10.8	131.6 ± 6.1	.404
Cl^−^ (98–107) (mmol/l)	101.2 ± 8.4	100.1 ± 7.6	.584
tCa^2+^ (8.6–10.2) (mg/dl)	9.0 ± 1.4	8.6 ± 1.1	.192
AST (<40) (Ul)	129.1 ± 467.3	138.6 ± 599.2	.939
CK (M: ∼39–308, F: ∼26–192) (U/l)	232.6 ± 360.0	371.0 ± 1028.1	.439
TnI (<40) (pg/ml)	219.2 ± 847.2	609.2 ± 1600.6	.202
proBNP (<125) (pg/mL)	6981.6 ± 7202.6	18 262.5 ± 14870.2	<.001
BUN (7–25) (mg/dl)	90.4 ± 50.0	131.4 ± 61.3	.003
Cr (M: 0.7–1.2, F: 0.5–0.9) (mg/dl)	5.4 ± 4.7	9.2 ± 5.9	.003
Alb (3.5–4.5) (g/dl)	3.1 ± 0.6	3.4 ± 0.5	.045
CRP (<0.8) (mg/dl)	6.5 ± 10.9	5.6 ± 7.8	.045
PCT (<0.05) (ng/ml)	3.0 ± 4.7	2.1 ± 5.0	.359
Echocardiography			
LVEF (50–70) (%)	60.3 ± 11.0	56.4 ± 16.4	.267

Abbreviation: ACEI, angiotensin-converting enzyme inhibitors; AI, artificial intelligence Alb, albumin AST, aspartate aminotransferase ARB, angiotensin receptor blockers; BMI, body mass index BUN, blood urea nitrogen CAD, coronary artery disease CHF, chronic heart failure Cl^−^, chloride COPD, chronic obstructive lung disease Cr, Creatinine F, female Hb, hemoglobin HCO_3_, bicarbonate HLP, hyperlipidemia HTN, hypertension LVEF, left ventricular ejection fraction M, male, potassium Na^+^, sodium ref, reference PCT, procalcitonin PLT, platelet ref., reference SBP, systolic blood pressure tCa, total calcium, TnI, troponin I WBC, white blood cells.

### Timing and difference of paired Lab-K^+^ and ECG-K^+^ measurements

**Figure 2:**
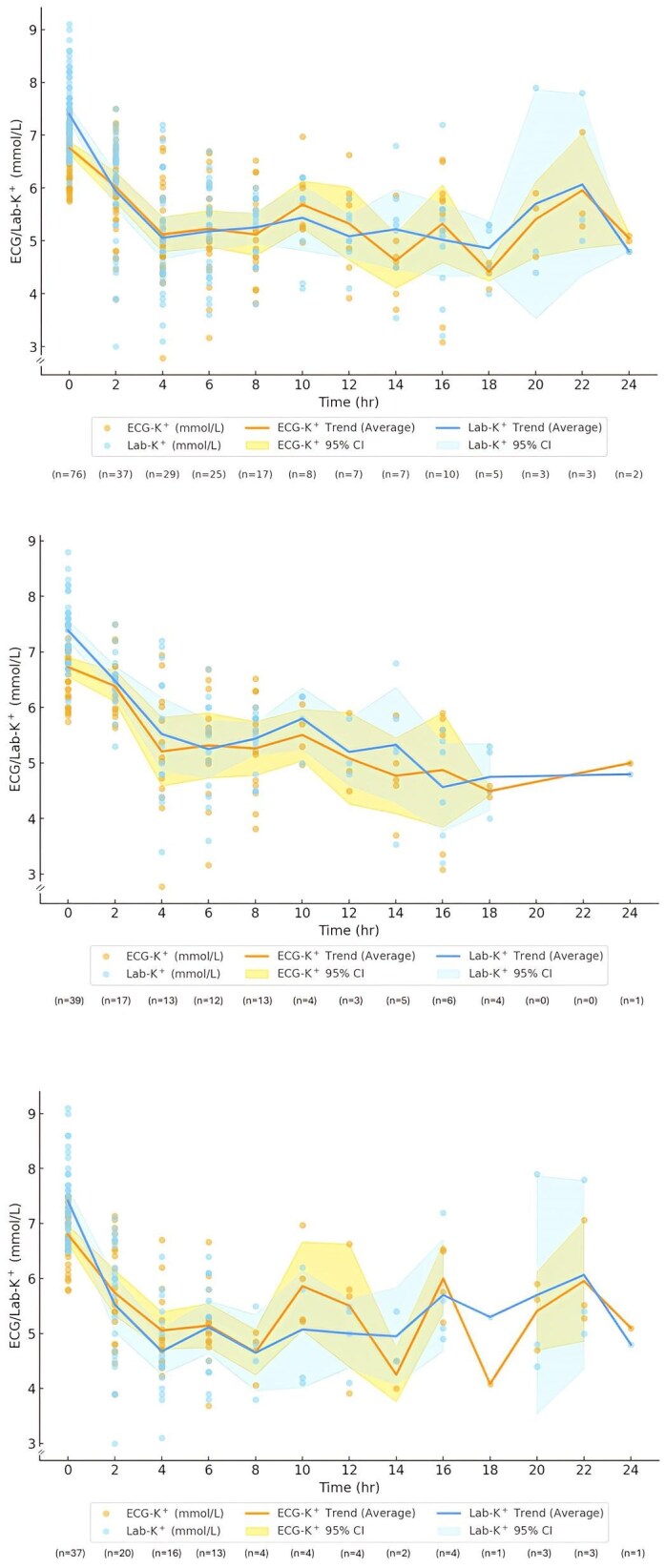
Time of paired AI-ECG-K^+^ and Lab-K^+^ during and after hyperkalemia treatment in all 76 patients (**a**), medication group (**b**), and HD group (**c**). Most paired ECG-K⁺ and Lab-K⁺ (70.6%) were performed within the first 8 hours in all 76 patients. (a) Within the first 4 hours, the HD group had 48% of paired ECG-K⁺ and Lab-K⁺, compared to 38.5% in the medication group. (b, c) A parallel decline trend in paired Lab-K⁺ and ECG-K⁺ levels was observed, particularly in the first 4 hours.

The mean time difference between Lab-K^+^ and ECG-K^+^ measurements during or after treatment was 11.4 ± 5.6 min. The detailed time course of paired ECG-K^+^ and Lab-K^+^ measurement was shown in Fig. 2. Most paired ECG-K^+^ and Lab-K^+^ (70.6%) were frequently performed within the first 8 hours in all 76 patients (Fig. 2a). Within the first 4 hours, 48% of paired ECG-K^+^ and Lab-K^+^ measurements were observed in the HD group, compared to 38.5% in the medication group (Fig. 2b, c). Despite variability, a parallel decline trend in both Lab-K^+^ and ECG-K^+^ levels was observed in all 76 patients, especially in the first 4–6 hours.

**Figure 3:**
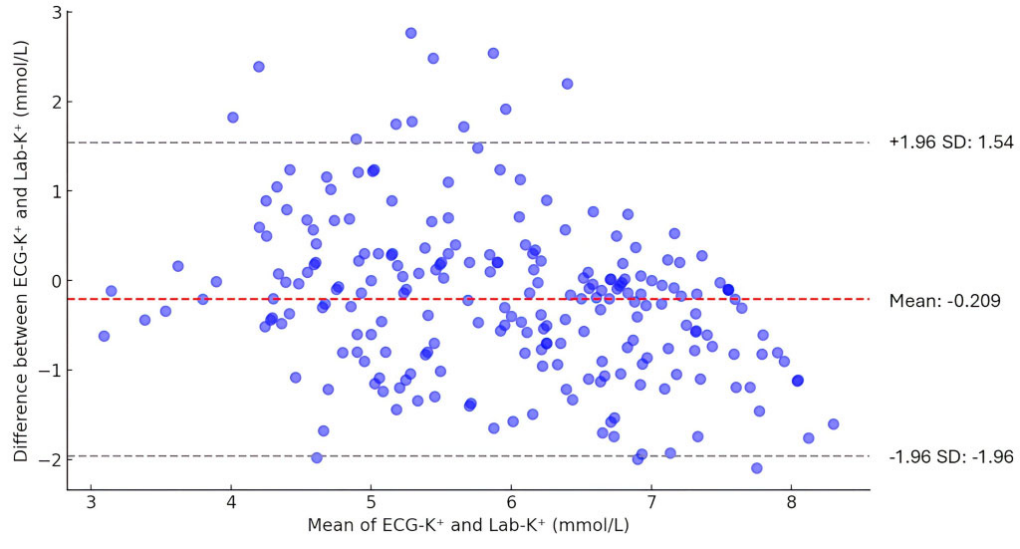
Bland and Altman plot between ECG-K^+^ and Lab-K^+^. The mean discrepancy between ECG-K⁺ and Lab-K⁺ was −0.209 mmol/l, indicating lower ECG-K⁺ readings, especially in severe hyperkalemia. The limits of agreement, which range from −1.96 to 1.54 mmol/l, encompass 95% of the variations, underscoring substantial concordance but also inherent variability between ECG-K⁺ and Lab-K⁺.

As illustrated in the Bland and Altman plot (Fig. 3), the mean difference between ECG-K^+^ and Lab-K^+^ was −0.209 mmol/l, suggesting ECG-K⁺ consistently records lower values than Lab-K⁺, a trend particularly pronounced in the presence of severe hyperkalemia. The limits of agreement ranging from −1.96 to 1.54 mmol/l captured 95% of the differences between ECG-K^+^ and Lab-K^+^, highlighting a general concordance yet intrinsic variability across two measurements.

### The changes in Lab-K^+^ and ECG-K^+^

The time difference between paired Lab-K^+^ and ECG-K^+^ was similar between time 1 and time 2–4 (*P *= .298). A significant and simultaneous decrease in both Lab-K^+^ and ECG-K^+^ was shown after hyperkalemia treatment (Supporting Fig. S2a). The degree of the followed Lab-K^+^ included 19.6% (30 of 153) severe hyperkalemia, 23.5% (36 of 153) moderate hyperkalemia, 19.0% (29 of 153) mild hyperkalemia, and 37.9% (58 of 153) of normokalaemia (*n* = 53) to mild hypokalemia (*n* = 5). As expected, a notable decrease in the number of severe hyperkalemia was found with sensitivity 86.7% and specificity 61.8% of paired AI-ECG for detecting severe hyperkalemia. For moderate hyperkalemia, the sensitivity and specificity were 83.3% and 70.1%, respectively, while for mild hyperkalemia, these values were 81.1% and 74.1%, respectively.

**Figure 4:**
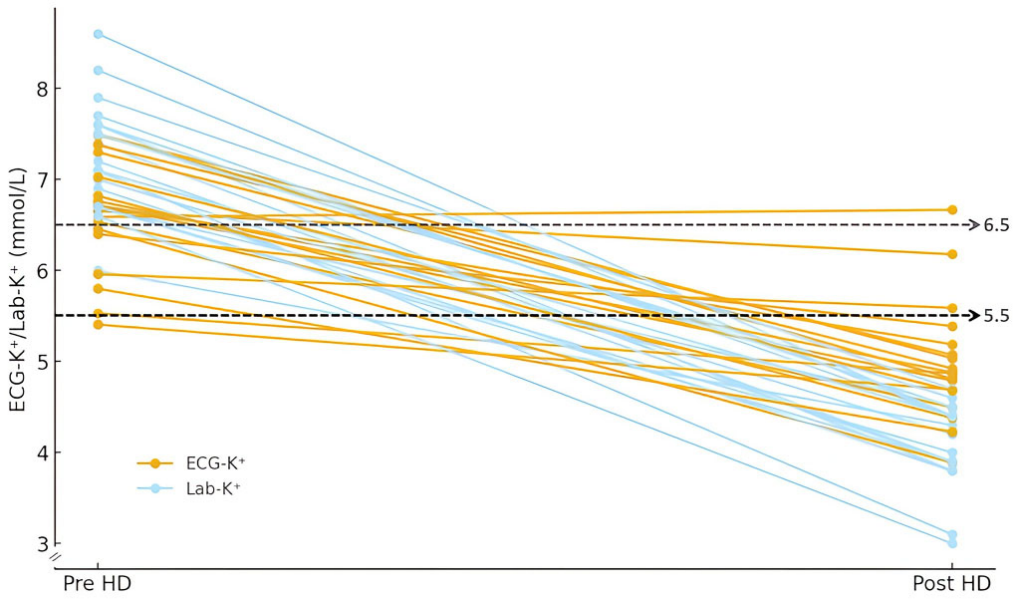
Changes of paired AI-ECG-K^+^ and Lab-K^+^ before and after the end of HD. There was significantly greater decline (7.3 to 4.1 mmol/l, a 3.2 mmol/L difference) in Lab-K⁺ shortly after the end of HD as compared to ECG-K⁺ (6.6 to 5.0 mmol/l, a 1.6 mmol/l difference). *The dotted lines were denoted for Lab-K^+^ ≥6.5 mmol/l and ECG-K^+^ ≥5.5 mmol/l.

The mean time duration for HD was 3.6 ± 0.8 hours. Most patients in the HD group received HD between time 1 to time 2 period (29 of 37, 78.4%). HD group patients achieved a more rapid decrease in Lab-K^+^ from 7.4 (95% CI 7.2–7.7) to 5.2 (95% CI 4.8–5.5) than ECG-K^+^ from 6.8 (95% CI 6.6–7.0) to 5.4 (95% CI 5.1–5.7) mmol/l levels over time 2 (Fig. S2B, C). In the patients receiving HD (*n* = 37), paired Lab-K^+^ and ECG-K^+^ before and after HD was 7.4 (95% CI 7.1–7.6) to 4.7 (95% CI 4.4–5.0) for Lab-K^+^ and 6.8 (95% CI 6.6–7.0) to 5.2 (95% CI 5.0–5.5) for ECG-K^+^, respectively (*P *< .001). Of interest, we also found that a more significant decrement in Lab-K⁺ from 7.3 (95% CI 6.9–7.6) to 4.1 (95% CI 3.9–4.3) mmol/l as compared to ECG-K⁺ from 6.6 (95% CI 6.3–6.9) to 5.0 (95% CI 4.5–5.3) mmol/l shortly (16.5 ± 11.1 minutes) after the end of HD in 18 patients (*P *< .001) (Fig. 4). Notably, two of them had persistent AI-ECG hyperkalemia following HD (see next).

### Persistent AI-ECG derived hyperkalemia

**Figure 5:**
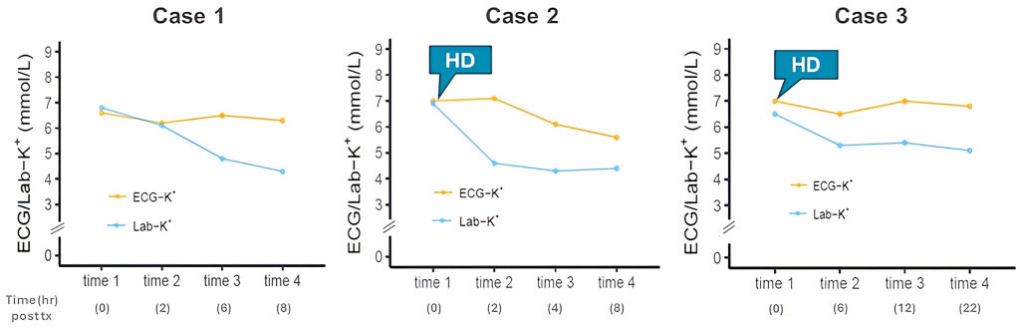
Persistent AI-ECG hyperkalemia despite normalization in Lab-K^+^ values in three patients.

Of interest, three (one with medication and two with HD) of 76 patients (3.9%) exhibited persistent AI-ECG hyperkalemia at least twice despite normalization in Lab-K^+^ values (Fig. 5). As shown in the Supplementary Table S1, they featured severe structural heart disease such as severely decompensated heart failure (*n* = 1), acute coronary syndrome (*n* = 2), and reduced left ventricular ejection fraction (37.5% ± 4.1%). More severe anemia (hemoglobin 8.0 ± 0.9 gm/dl), hyponatremia, hypocalcemia, and metabolic acidosis were also common. Persistent AI-ECG hyperkalemia in all three patients were normalized within 2 weeks after their underlying acute coronary syndrome and heart failure subsided.

## DISCUSSION

It is crucial to monitor serum K^+^ levels and ECG changes during the treatment of severe hyperkalemia. This retrospective study was to evaluate the role our AI-ECG-K^+^ prediction in 76 patients with at least two paired Lab-K^+^ and ECG-K^+^ during and after the treatment of severe hyperkalemia. We found that most of them had both Lab-K^+^ and ECG-K^+^ significantly declined in parallel, either treated medically (*n* = 39) or with HD group (*n* = 37). However, three patients exhibiting persistent ECG-K^+^ hyperkalemia despite the normalized Lab-K^+^ levels had acute or severe concomitant cardiovascular comorbidities. To the best of our knowledge, this was the first study to investigate the quantitative AI-ECG-K^+^ in monitoring true severe hyperkalemia.

Given a much higher risk of cardiac arrhythmia in patients with hyperkalemia, a Kidney Disease: Improving Global Outcomes (KDIGO) Controversies Conference has recommended to evaluate both the severity of Lab-K^+^ hyperkalemia and its associated ECG changes as a guide for management [3]. Serum Lab-K^+^ ≥ 6.5 mmol/l regardless of ECG changes and Lab-K^+^ ≥ 6.0 mmol/l with ECG alterations are indicative of urgent serum K^+^-lowering therapy [3]. Several authoritative guidelines also advocate frequent 12-lead ECG reassessments along with Lab-K^+^ measurement during the treatment of acute hyperkalemia [3, 22, 24, 25]. Since the sensitivity of physician ECG interpretation to recognize hyperkalemic ECG changes was relatively low [12], our AI-ECG model for prompt and quantitative hyperkalemia detection with much higher sensitivity than that by physicians may be used to help monitor serum K^+^ changes during the hyperkalemia treatment but should still be evaluated in this study [21].

With paired Lab-K^+^ and ECG-K^+^ during and after hyperkalemia treatment in this study, we found that both Lab-K^+^ and ECG-K^+^ significantly declined in parallel among medication and HD group patients. The sensitivity and specificity of AI-ECG-K^+^ for detecting severe hyperkalemia was 86.7% and 61.8%, respectively, which was lower compared to our previous cohort studies (93.8% and 91.8%) focusing primarily on initial hyperkalemia detection [21]. The medications such as intravenous calcium administration to counteract cardiac toxicity of severe hyperkalemia via different mechanisms rather than directly lowering Lab-K^+^ levels might contribute to an increase in false negatives with a decrease sensitivity [26]. The markedly decreased number of severe hyperkalemia (19.6%, 30 of 153) might also cause the reduced sensitivity of AI-ECG-K^+^ monitoring. Owing to the underlying medical complexity in these patients with severe hyperkalemia, we found that there was a higher false positive rate of AI-ECG monitoring for hyperkalemia, which might lead to inconvenience and anxiety for clinicians. However, pseudo-positive EKG-K^+^ for hyperkalemia has been shown to importantly predict all-cause mortality, akin to the concept of a “previvor” [21].

Of interest, we found that a greater decrease in post-HD Lab-K^+^ than ECG-K^+^ in all patients receiving HD. In a subset of patients (48.6%, 18 of 37) who had paired data shortly after HD, they exhibited a much greater decline in Lab-K^+^ as compared to ECG-K⁺. The reasons for the larger difference between post-HD ECG-K⁺ and Lab-K^+^ remain elusive. It is known that HD can effectively reduce severe hyperkalemia within 2 to 4 hours via rapid diffusion along the blood-dialysate K^+^ gradient [27, 28]. Although normalized Lab-K⁺ levels after HD may attenuate the higher resting membrane potential and cardiac excitability associated with hyperkalemia [29–33], adaptations in cardiac cellular ion channels, such as the Na⁺/K⁺ pump in response to extracellular K⁺ changes, can be delayed [34]. Since diffusion of cardiac intracellular K⁺ into blood may be slower than that in blood-dialysate K⁺ gradient, lower dialysate K⁺ concentrations of 1.0 mmol/l used in this study to achieve a more rapid serum K⁺ reduction may account for a higher post-HD ECG-K⁺ values. Based on the delayed recovery of post-HD hyperkalemic ECG-K⁺, it is possible that the use of dialysate K^+^ concentration 2.0 mmol/l or higher even in severe hyperkalemia may reduce this potentially detrimental cardiac effect, although further investigation is warranted [10].

Unlike transient hyperkalemic AI-ECG after HD, we observed three patients with persistent AI-ECG hyperkalemia at least twice despite normalized serum K^+^ levels after treatment. They exhibited more severe underlying patients’ factors such as acute coronary syndrome, heart failure, coexisting severe metabolic acidosis, hyponatremia, hypocalcemia, and anemia. These non-hyperkalemic cardiac and non-cardiac conditions might adversely affect cardiac excitability and conduction velocity, causing similar ECG features of hyperkalemia [6, 35–37]. Like cardiac troponin as a reflection of myocardial injury, the ECG signatures of hyperkalemia may flag a poorer prognosis, which necessitate more attention to correct the underlying coexisting illness. Of interest, persistent ECG-K^+^ hyperkalemia appeared to be reversible within weeks once these underlying problems were corrected. It was also possible that patients with persistent ECG-K^+^ hyperkalemia may exhibit a diminished arrhythmogenic threshold, requiring consistent monitoring to prevent adverse outcomes. Given a higher false-positivity of AI-ECG in hyperkalemic patients, we believed that persistent AI-ECG hyperkalemia may be underestimated, as only a limited number of ECGs were conducted after serum K^+^ levels were normalized after treatment.

There were some limitations to this study. First, our trained AI-ECG model for quantitative hyperkalemia exhibited a more restricted range (5.5 to 7.5 mmol/l) compared to Lab-K^+^ (5.5 to 10.0 mmol/l). Consequently, the threshold for severe hyperkalemia using ECG-K^+^ (≥5.5 mmol/l) was set lower than that for Lab-K^+^ (≥6.5 mmol/l). This discrepancy also imposed limitations on the AI-ECG model's ability to estimate ECG-K^+^ in patients presenting with extremely high Lab-K^+^ levels (≥7.5 mmol/l). Second, the selection bias caused by the exclusion of severe hyperkalemic patients with initially matched ECG-K^+^ <5.5 mmol/l (6.3%, 22 of 348), and infrequently matched AI-ECG-K^+^ and Lab-K^+^ measurement during hyperkalemia treatment, may decrease the generalizability of our results. Third, the small sample size (*n* = 76) that diminished the study's statistical power also limited the generalizability of our findings. Fourth, the impact of different dialysate K⁺ concentrations on ECG-K⁺ changes and associated cardiac risk in severe hyperkalemia was not evaluated in this study. Fifth, retrospective design of this study inevitably resulted in a wide variety of time intervals in different paired Lab-K^+^/ECG-K^+^ measurements; prospective studies are warranted to validate our findings. Finally, our aim was to assess the role of AI-ECG in monitoring severe hyperkalemia rather than its utility as a decision support tool in real-world scenarios. The lead time of ECG-K^+^ over Lab-K^+^, ∼30–40 min, may still offer the potential advantage for treating severe hyperkalemia [3, 38, 39].

In summary, this study demonstrated the effectiveness of real-time AI-ECG-K^+^ in monitoring serum K^+^ levels for patients with true severe hyperkalemia. Two additional novel findings included a significantly greater decline in Lab-K⁺ than ECG-K⁺ shortly after HD, and persistent ECG-K^+^ hyperkalemia despite the normalized Lab-K^+^ in few patients accompanied by acute severe cardiovascular comorbidities and acid-base/electrolytes imbalance. A large prospective clinical study using Lab-K⁺ and AI-ECG-K⁺ is still warranted to validate our findings in the patients with severe hyperkalemia.

## Supplementary Material

sfaf092_Supplemental_File

## Data Availability

All data generated or analyzed during this study are included in this article. Further inquiries can be directed to the corresponding author.
