# Supplementary material for: Monitoring serum potassium concentration in patients with severe hyperkalemia: the role of bloodless artificial intelligence-enabled electrocardiography
Source: Clin Kidney J. 2025 Apr 8;18(4):sfaf092. doi: 10.1093/ckj/sfaf092 (PMC12032525; doi:10.1093/ckj/sfaf092)

Table S1. Clinical characteristics of the patients with persistent AI-ECG-hyperkalemia

| **Patients** | **1** | | | | **2** | | **3** | |
| --- | --- | --- | --- | --- | --- | --- | --- | --- |
| **Treatment** | **Med** | | | | **HD** | | **HD** | |
| **Demography** |  |  |  |  |  |  |  |  |
| Age (yrs) | 83 | | | | 32 | | 88 | |
| Sex | M | | | | M | | F | |
| SBP | 126 | | | | 95 | | 147 | |
| **General comorbidities** | DM  CAD | | | | DM  CAD | | DM  CHF | |
| **Kidney-specific comorbidities** | AoCKD | | | | ESKDd | | ESKDd | |
| **Lab test** (ref range) |  | | | | | | | |
| WBC (4.5-11.0) (10^3^/ul) | | 8470 | 16120 | | | 9550 | | |
| Hb (M:13.5~18.0, F:12.0~16.0) (gm/dL) | | 8.4 | 8.8 | | | 6.7 | | |
| Plt (150-400) (10^3^/ul) | | 292 | 292 | | | 153 | | |
| pH (7.35-7.45) | | 7.24 | 7.16 | | | 7.18 | | |
| HCO_3_^-^ (23-27) (mmol/L) | | 17 | 7.1 | | | 13.2 | | |
| Na^+^ (135-145) (mmol/L) | | 132 | 133 | | | 124 | | |
| K^+^ (3.5-4.5) (mmol/L) | | 6.8 | 6.9 | | | 6.5 | | |
| Cl^-^ (98-107) (mmol/L) | | 110 | 98 | | | 89 | | |
| CK (M:39~308, F:26~192) (U/L) | | 1169 | 6057 | | | 266 | | |
| TnI (<40) (pg/mL) | | 42 | 118 | | | 32 | | |
| tCa^2+^ (8.6-10.2) (mg/dL) | | 7.1 | 7.8 | | | 7.3 | | |
| proBNP (<125) (pg/mL) | | 1822 | 28601 | | | 35000 | | |
| BUN (7-25) (mg/dL) | | 72 | 231 | | | 126 | | |
| Cr (M: 0.7-1.2, F: 0.5-0.9) (mg/dL) | | 4.6 | 25 | | | 7.1 | | |
| Alb (3.5-4.5) (g/dL) | | 3.4 | 3.0 | | | 3.2 | | |
| CRP (<0.8) (mg/dL) | | 0.21 | 1.24 | | | 8.21 | | |
| PCT (<0.05) (ng/mL) | | NA | NA | | | 1.58 | | |
| **Echocardiography** |  |  |  |  |  |  |  |  |
| LVEF (%) | 40-45 | | | 30-40 | | | | 30-35 |
| **Progression** |  | | | | | | | |
| First normalized AI-ECG-K^+^  (ECG-K^+^ level, days after index time) | (4.6, 5) | | | (4.3, 3) | | | | (5.0, 12) |
| Abbreviation: aCKD, advanced chronic kidney disease, AI, aritificial intelligence, AKI, acute kidney injury, Alb, albumin, AST, aspartate aminotransferase, BMI, body mass index, BUN, blood urea nitrogen, CAD, coronary artery disease, CHF, chronic heart failure, Cl-, chloride, COPD, chronic obstructive lung disease, Cr, Creatinine, CRP, C-reactive protein, DM, diabetes mellitus, ECG, echocardiography, end stage kidney disease on dialysis, ESKDd, GIB, gastrointestinal bleeding, HD, hemodialysis group, Hb, hemoglobin, HCO3, bicarbonate, HLP, hyperlipidemia, HTN, hypertension, LVEF, left ventricular ejection fraction, Med, medication group, Non-dialysis dependent chronic kidney disease, ref, reference, PCT, procalcitonin, PLT, platelet, proBNP, pro-B type natriuretic peptide, SBP, systolic blood pressure, tCa, total calcium, TnI, troponin I, WBC, white blood cells | | | | | | | | |

**Figure S1. Visualized AI-ECG based analysis for hyperkalemia in a 12-lead ECG format**


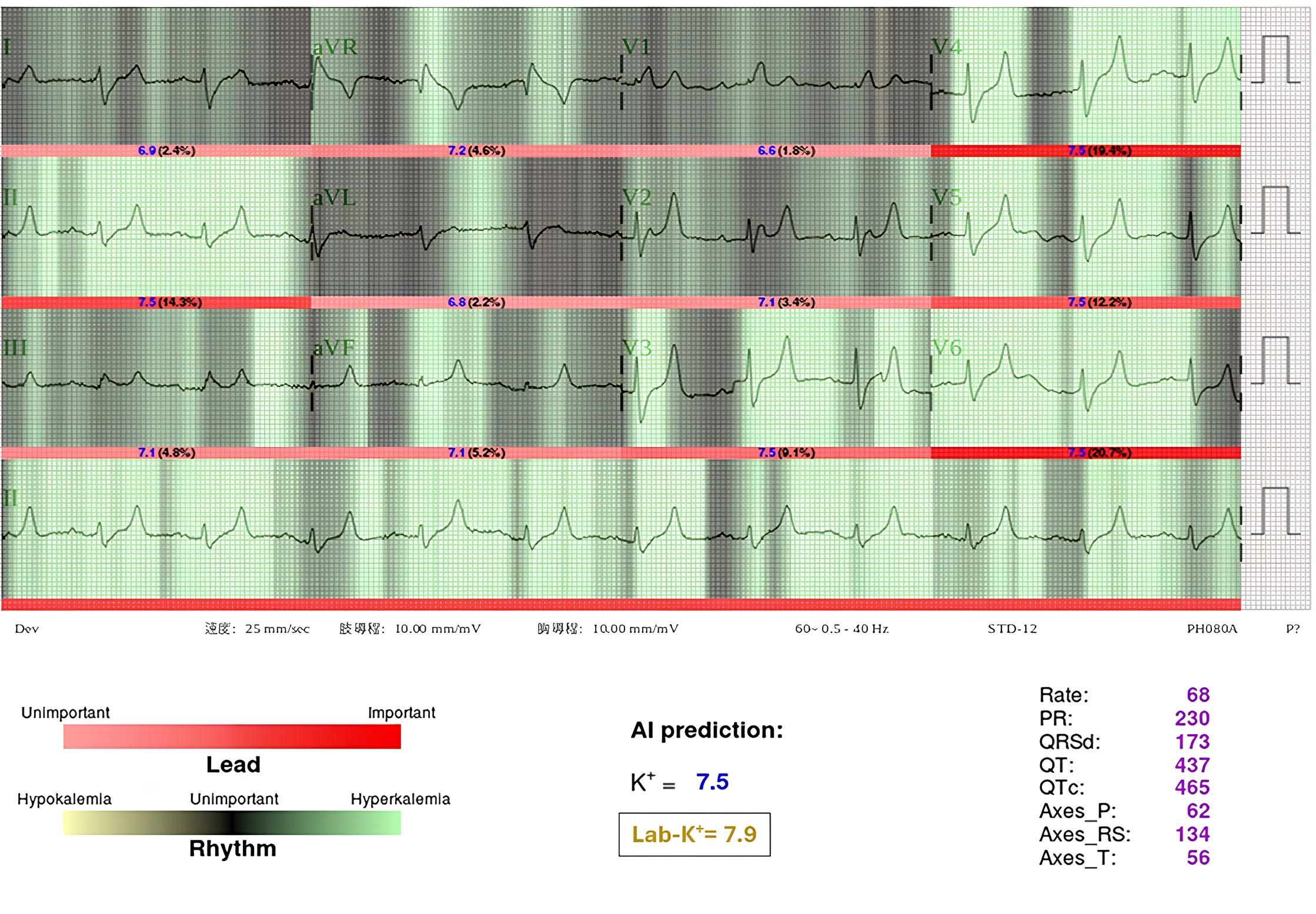


**Figure S2.** **Paired AI-ECG K^+^ & Lab-K^+^ during and after hyperkalemia treatment in all 76 patients (A), medication group (B), and HD group (C)**


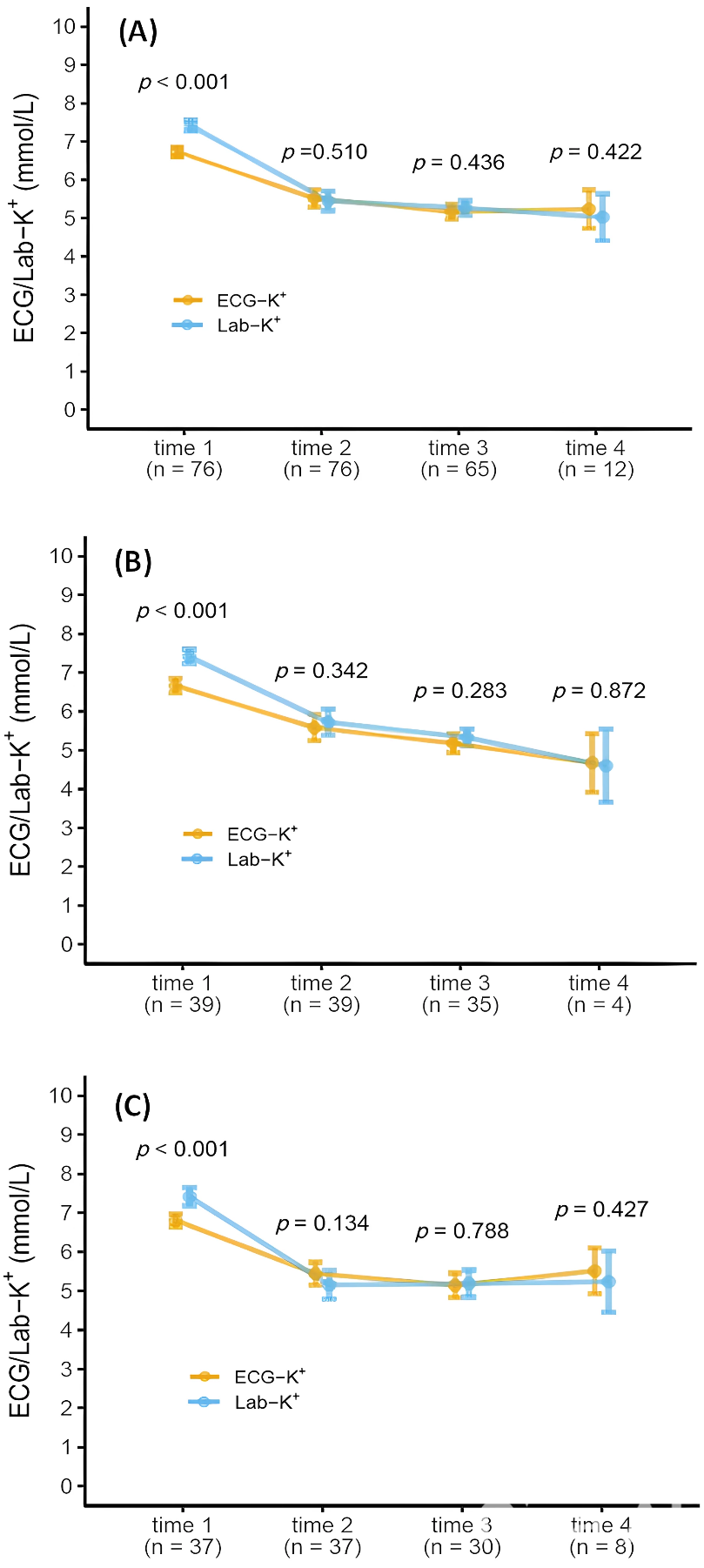

Supplement: sfaf092_Supplemental_File [file sfaf092_supplemental_file.docx]
